# Differences in number and distribution of striatal calbindin medium spiny neurons between a vocal-learner (*Melopsittacus undulatus*) and a non-vocal learner bird (*Colinus virginianus*)

**DOI:** 10.3389/fnana.2013.00046

**Published:** 2013-12-19

**Authors:** Elena Garcia-Calero, Olga Bahamonde, Salvador Martinez

**Affiliations:** ^1^Department of Experimental Embryology, Instituto de Neurociencias, Universidad Miguel Hernández-Consejo Superior de Investigaciones CientíficasSan Juan, Alicante, Spain; ^2^Fundación Investigación Clínico de Valencia-Instituto de Investigación SanitariaValencia, Spain

**Keywords:** striatum, evolution, song system, language learning, FoxP1, subventricular zone

## Abstract

Striatal projecting neurons, known as medium spiny neurons (MSNs), segregate into two compartments called matrix and striosome in the mammalian striatum. The matrix domain is characterized by the presence of calbindin immunopositive (CB+) MSNs, not observed in the striosome subdivision. The existence of a similar CB+ MSN population has recently been described in two striatal structures in male zebra finch (a vocal learner bird): the striatal capsule and the Area X, a nucleus implicated in song learning. Female zebra finches show a similar pattern of CB+ MSNs than males in the developing striatum but loose these cells in juveniles and adult stages. In the present work we analyzed the existence and allocation of CB+ MSNs in the striatal domain of the vocal learner bird budgerigar (representative of psittaciformes order) and the non-vocal learner bird quail (representative of galliformes order). We studied the co-localization of CB protein with FoxP1, a transcription factor expressed in vertebrate striatal MSNs. We observed CB+ MSNs in the medial striatal domain of adult male and female budgerigars, although this cell type was missing in the potentially homologous nucleus for Area X in budgerigar. In quail, we observed CB+ cells in the striatal domain at developmental and adult stages but they did not co-localize with the MSN marker FoxP1. We also described the existence of the CB+ striatal capsule in budgerigar and quail and compared these results with the CB+ striatal capsule observed in juvenile zebra finches. Together, these results point out important differences in CB+ MSN distribution between two representative species of vocal learner and non-vocal learner avian orders (respectively the budgerigar and the quail), but also between close vocal learner bird families.

## INTRODUCTION

Medium spiny neurons integrate cortico-basal ganglia-thalamo-cortical circuits for motor learning in vertebrates (Graybiel et al., 1994; Packard and Knowlton, 2002; Fino and Venance, 2010; review of MSN circuits in vertebrates in Reiner et al., 1998). Vocal acquisition by imitation is an example of motor learning, described in mammals like humans, cetaceans and bats, and in vocal learner birds like songbirds (passerines), parrots, and hummingbirds (i.e., whales: Payne and McVay, 1971; dolphins: Richards et al., 1984; Janik, 2000; bats: Esser, 1994; songbirds: Thorpe, 1958; parrots: Farabaugh et al., 1994; Heaton and Brauth, 1999; hummingbirds: Baptista and Schuchmann, 1990). Vocal learner birds develop a special neural circuit for song learning and production, the song system. This so called song system depends on MSN function in its striatal subdivision (Nottebohm and Arnold, 1976; Nottebohm et al., 1976; Striedter, 1994; Durand et al., 1997; Gahr, 2000; Roberts et al., 2002). Other birds, like chickens or quails, only produce innate sounds and they do not develop a network of telencephalic nuclei for vocal learning (Konishi, 1963; Gahr, 2000; Puelles et al., 2007). In addition, parrots, the proposed closest living relatives of passerines (Suh et al., 2011) are also able of movement learning by imitation (Moore, 1992), which, like song learning, implicates striatal projecting neuron circuits.

During the development of the mammalian striatum MSNs segregate into two main compartments: striosome and matrix (Gerfen et al., 1985; Gerfen, 1992; Liu and Graybiel, 1992b; Crittenden and Graybiel, 2011). Striatal compartmentalization has a functional implication. While MSNs in the matrix domain participate in sensorimotor and associative circuits, the striosome MSNs are involved in the limbic system (Jimenez-Castellanos and Graybiel, 1987; Gerfen, 1989; Eblen and Graybiel, 1995; Kincaid and Wilson, 1996; reviewed in Crittenden and Graybiel, 2011). The matrix domain originates late during development from the subventricular zone (SVZ) and is characterized by the presence of CB+ MSNs (Gerfen et al., 1985; van der Kooy and Fishell, 1987; Liu and Graybiel, 1992b; Anderson et al., 1997; Garel et al., 1999; Mason et al., 2005). Our recent results show the existence of a population of CB+ MSNs in the striatal domain of male zebra finch (Garcia-Calero and Scharff, 2013). These cells concentrate in the striatal capsule and Area X, a song system nucleus that participates in learning and production of song in songbirds (Sohrabji et al., 1990; Scharff and Nottebohm, 1991; Jarvis and Nottebohm, 1997; Jarvis et al., 1998; Hessler and Doupe, 1999). These data suggest a role of CB+ MSNs in Area X function. In female zebra finches a similar CB+ MSN population appears during development, however, from PHD20 onward these neurons are no longer observed in the Area X, although remain present in the CB+ striatal capsule (Garcia-Calero and Scharff, 2013). Our previous work links this event to differences in HVC inputs arrival mediated by sexual hormones. HVC is another nucleus of the song system located in the pallial subdivision that receives auditory information from Field L and projects to different nuclei of the song system like the Area X or the robust nucleus in the arcopallium (Nottebohm et al., 1976; Kelley and Nottebohm, 1979).

In contrast to songbirds, the existence of a CB+ MSN population in the striatal domain of parrots remains unclear, even in the magnocellular nucleus of the medial striatum (MStm), which has been described as a nucleus potentially homologous to Area X (Striedter, 1994; Durand et al., 1997; Reiner et al., 2004). In non-vocal learner birds a nucleus similar to Area X is not detectable in the striatal region and the presence of a striatal CB+ MSN population is not yet proven (Bálint and Csillag, 2007; Husband and Shimizu, 2011).

In this work we analyzed the presence of CB+ MSNs in the striatal domain of the vocal learner bird budgerigar (psittaciformes order) and the non-vocal learner bird quail (galliformes order), to further characterize the differences of CB+ MSN distribution in the striatal domain between these two avian orders and its relation with motor learning capabilities. We studied the co-localization of CB with FoxP1, a marker for MSNs in vertebrates (Tamura et al., 2003; Haesler et al., 2004; Teramitsu et al., 2004) and analyzed the cellular distribution of CB+ cells in the striatal domain of male and female budgerigars to better characterize the gender differences that have been previously described in these birds (Brauth et al., 2005). Finally, following the recent description of a CB+ striatal capsule in zebra finch (Garcia-Calero and Scharff, 2013), we analyzed the presence of this neuroanatomical structure in budgerigar and quail striatum. We observed CB+ MSNs in adult male/female budgerigar striatum although the distribution appears different than in male zebra finches; in contrast, there were no CB+ MSNs in adult male quail striatum. We also analyzed the presence of CB+ cells in quail striatum at developmental stages to compare the results to the previous description of this cell type in female zebra finches during development but not at later stages (Garcia-Calero and Scharff, 2013). In addition, we observed the existence of a CB+ striatal capsule in budgerigar but not in quail striatum.

## MATERIALS AND METHODS

The animals were treated according to the regulations and laws of the European Union (86/609/EEC) and the Spanish Government (Royal Decree 223/1998) for care and handling of animals in research.

### TISSUE PREPARATION

We obtained quail embryos from fertile quail eggs collected from domestic quails (*Colinus virginianus*) from local breeders. Collected eggs were transferred to an egg incubator at 37.8°C and 50–60% humidity. Adult quail, budgerigars (*Melopsittacus undulatus*), and juvenile zebra finches (*Taeniopygia guttata*) were also obtained from local breeders. The embryos were anesthetized on ice prior to sacrifice and the brains were dissected out and fixed overnight in 4% paraformaldehyde in pH 7.4 phosphate-buffered saline (PBS) at 4°C. For adult quail, budgerigars, and juvenile zebra finches, animals were overdosed with isoflurane and subsequently perfused transcardially with the same fixative solution as above and postfixed for 24 h at 4°C. The tissue was embedded in 4% agarose in PBS and 50 μm sections were cut in horizontal planes with a Leica vibratome (VT1000 S), to be processed for immunohistochemistry or for cresyl-violet staining.

### IMMUNOHISTOCHEMISTRY

For immunostaining, the sections were treated with 0.3% hydrogen peroxide in PBS+ 0.3% Triton (PBT) for 15 min to inactivate endogenous peroxidase activity. After several washes in PBT, sections were blocked in PBS containing 0.3% Triton X-100 and 3% BSA, and incubated in the primary antiserum for 2 days at 4°C. Following this incubation and standard washes in PBT, the sections were incubated in a secondary biotinylated antiserum for 2 h at room temperature (RT; Vector, Burlingame, CA, USA). After washing, the sections were incubated in avidin-biotin complex (ABC kit; Vector; 0.003% dilution) for 1 RT. The immunolabeling was revealed by 0.05% diaminobenzidine (DAB; Sigma-Aldrich, Steinheim, Germany) in 0.05 M Tris buffer (pH 7.6), containing 0.03% H_2_O_2_. The following primary antibodies were used: rabbit anti-Calbindin (Swant); mouse anti-FoxP1 (Abcam). For immunofluorescence staining, appropriate secondary antibodies coupled to fluorescent dyes were used: anti-rabbit Alexa 488, anti-mouse Alexa 594 (Molecular Probes Europe BV, Leiden, Netherlands, 1:200). For control of immunohistochemistry we prepared negative control sections by leaving out the primary antibody; these control sections showed no staining.

### ANTIBODY CHARACTERIZATION

Calbindin (rabbit anti-Calbindin, Swant; Bellinzona, Switzerland, dilution 1:1000). The CB polyclonal antibody detects a single band in Western blots of chick brain tissue (Suarez et al., 2006). Controls made by Suarez et al. (2006) incubating brain sections with the primary antibody pre-adsorbed with the immunizing peptide (1 mg of the recombinant protein for 1 ml of the diluted antibody) did eliminate staining. This antibody was also used previously in zebra finch (Heyers et al., 2008).

FoxP1 (mouse anti-FoxP1, ab-32010, Abcam, Cambridge, MA, USA, dilution 1:2000). The monoclonal antibody against FoxP1 detects the full length native protein (purified) of mouse and recognizes FoxP1 protein in MSNs of mouse striatal domain (Banham et al., 2001; Arlotta et al., 2008). The FoxP1 monoclonal antibody detects a single band in Western blots of Hek cells overexpressing zebra finch FoxP1 (Abcam, datasheet). This antibody does not recognize closely related molecules FOXP2, FOXP3, or FOXP4.

### IMAGE CAPTURE, MANIPULATION, AND FIGURE ASSEMBLY

Digital photomicrographs were obtained with digital camera DC500 or DC350 (Leica, Wetzlar, Germany) and Leica TCS-NT confocal microscope. Digital images were processed for contrast and brightness with Photoshop software (Adobe Systems Mountain View, CA, USA).

### QUANTIFICATION

For the quantification of co-localization patterns (CB/FoxP1), confocal images were analyzed using ImageJ (NIH, ) software. Double-labeled cells were counted from four different rostrocaudal levels of the striatal domain of two adult male budgerigars, one adult female budgerigar, one adult male quail, and three quail embryos. Data were expressed as average ± STD.

## RESULTS

### CB+ MSN IN THE STRIATAL DOMAIN OF MALE/FEMALE ADULT BUDGERIGAR

**FIGURE 1:**
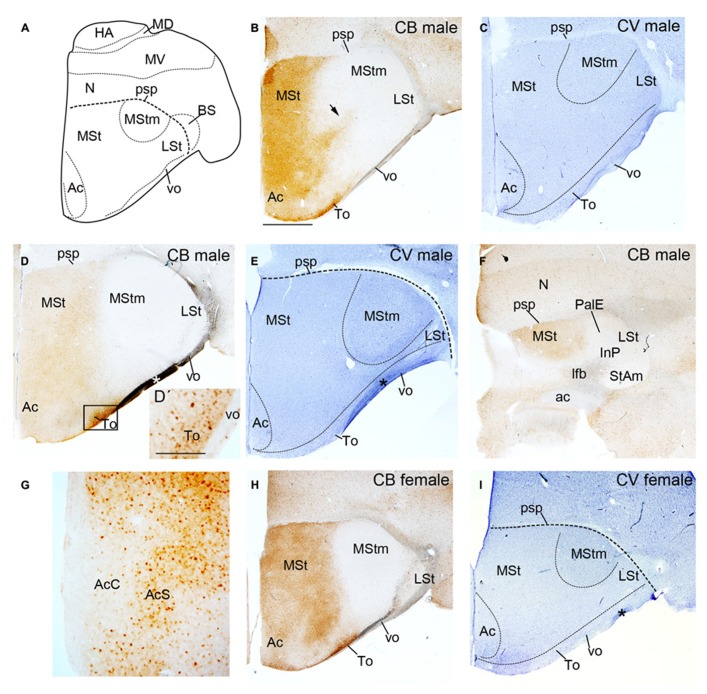
**Diagram of budgerigar telencephalon (A) and coronal sections through the striatum of adult male (B–G) and female (H,I) budgerigars showing CB immunostaining (B,D,D′,F–H) and cresyl-violet staning (C,E,I) from rostral to caudal levels**. The respective staining and gender are indicated at the upper right-hand corner of each panel. Dorsal is oriented toward the top of the photos, medial (bordering the ventricle) to the left. Black arrow in **(B)** point to the CB+ stream delimiting the MStm. Black rectangle in **(D)** delineates the region magnified in **(D′**). The asterisk in D, E and I indicates an artifact (fold at brain surface). For abbreviations, see list. Scale bar = 2.5 mm in **(B–D,E,F,H,I)**; 0.25 mm in **(D′,G)**.

**FIGURE 2:**
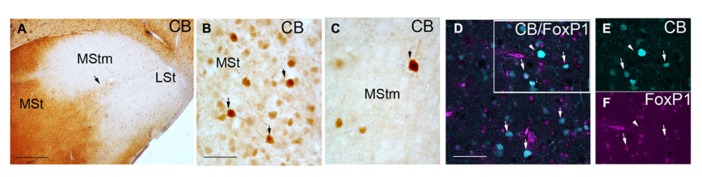
**Coronal sections through the striatum of adult male budgerigar showing CB immunostaining (A–C) and immunofluoresecence of CB in cyan and FoxP1 in magenta (D–F).** The respective staining is indicated at the upper right-hand corner of each panel. Dorsal is oriented toward the top of the photos, medial (bordering the ventricle) to the left. Black arrow in **(A)** point the CB+ stream delimiting the MStm. Black arrows in **(B,C)** point to CB+ cells with high immunoreactivity. White rectangle in **(D)** delineates the region magnified in **(E,F)**. White arrows in **(D–F)** indicate cells with co-localization of CB and FoxP1; white arrow head in **(D–F)** indicates a CB+ but FoxP1 negative cell. For abbreviations, see list. Scale bar = 2 mm in **(A)**; 62.5 μm in **(B,C)**; 31.25 μm in **(D–F)**.

We analyzed the presence of CB+ MSNs in the adult male (*n* = 2) and female (*n* = 1) budgerigar striatum, from rostral to caudal levels (**Figures 1** and **2**, where **Figure 1A**: diagram of budgerigar telencephalon showing the main neuronatomical subdivisions). We show cresyl-violet staining in parallel to our immunohistochemistry preparations for comparative purposes (**Figures 1C,E,I**). In our description we used the striatal subdivisions proposed by Puelles et al. (2007). The striatum is subdivided in two distinct radial subdomains. The most dorsal sector is close to the pallial /subpallial border (psp) and it contains the medial striatum (MSt) in the periventricular zone, and the lateral striatum (LSt) in the intermediate stratum. This domain finishes caudally in the striatoamygdaloid transition area (StAm). The striatal capsule overlaying the striatum is described in a different section of the present work. The second subdomain, adjacent to the pallidal region, is called the striatopallidal area (StPal) in Puelles et al. (2007). This field encloses the periventricular part of the StPal, the intrapeduncular nucleus (InP), and ends caudally in the striatopallidal amygdala (StPalA). The periventricular part of the StPal domain was not clearly detected in our cresyl violet and CB stainings and we decided to include it in the MSt as it has been done in previous studies in parrots (i.e., Roberts et al., 2002). We also examined the olfactory tubercle (To) extending along the striatal surface of the two radial domains. The accumbens nucleus (Ac) appears surrounding the ventral horn of the lateral ventricle in the most rostral sections.

The CB immunostaining was similar in male and female budgerigars, with minor differences in the area occupied by the vocal nucleus MStm (i.e., **Figures 1D,H**; gender differences in MStm size were previously reported in Brauth et al., 2005). We observed CB+ cells in the medial region of the striatum in male and female budgerigars; the lateral part of the striatum was almost devoid of CB immunoreactivity, although not completely (**Figures 1B,D,F,G,H** and 2). The CB+ medial striatal area encompassed the MSt and the nucleus Ac (**Figure 1**). In addition, in our most rostral sections, there was a strip of CB labeled cells extending from the medial striatal domain to more lateral regions (black arrow in **Figures 1B** and **2A**). This CB positive band delineated roughly the anterior and lateral edges of the MStm (compare with cresyl-violet in **Figure 1C**). In parrots, neurochemical studies (Durand et al., 1998; Roberts et al., 2002; Feenders et al., 2008) and zenk (an activity-dependent immediate early gene) upregulation during vocal activities (Jarvis and Mello, 2000; Brauth et al., 2002; Feenders et al., 2008) have been previously used to describe the MStm. This vocal nucleus, which is part of the medial striatum, was poor in CB immunoreactive cells (**Figures 1B,D,H** and **2A,C**). This result contrasts with the previous data published in zebra finch by Garcia-Calero and Scharff (2013). In addition, the Ac in the medial area of the striatal domain showed a clear distinction between the core and the shell subdivisions of this nucleus as it was previously reported in the budgerigar by Roberts et al. (2002) and in chicken (Bálint and Csillag, 2007; Garcia-Calero and Puelles, 2009). The core region was devoid of CB+ cells in contrast to the shell region (**Figure 1G**).

The lateral area although almost devoid of CB+ cells in both male and female budgerigars (**Figure 1**), still presented some scattered positive cells (**Figure 2A**). This region includes the classical LSt, which ends caudally in the the StAm, and was also poor in CB immunopositive cells (**Figures 1B,D,F,H**). The InP, immersed in the lateral forebrain bundle (lfb) mainly lacked CB+ cells (**Figure 1F**). The To extends in the pial surface covering the striatal domain and in contrast to the other lateral structures described before, this nucleus showed a rich population of CB+ cells (**Figures 1B,D,D′,H**).

We studied our CB preparations according to the type of CB+ cell labeled in male and female budgerigars. To date two different types of CB+ neurons have been described in the mouse striatal domain. One is dispersed in the striatum, with high CB immunoreactivity and corresponds to interneurons (Bennett and Bolam, 1993). The second type is located in the matrix domain and shows weak CB staining. This second group corresponds to projecting MSNs (Bennett and Bolam, 1993). In agreement with this study, Garcia-Calero and Scharff (2013) found these two different CB+ cell populations in male Area X of zebra finch. We observed some scattered heavily labeled CB+ cells (some example pointed with black arrows in **Figures 2B,C**) whereas most of cells showed weaker CB signal in budgerigar striatum. We analyzed the co-localization of CB protein with FoxP1, a transcription factor expressed in vertebrate striatal MSNs but not in interneurons (Haesler et al., 2004; Tamura et al., 2004; Teramitsu et al., 2004), to distinguish the two types of CB+ neurons described before. The vast majority of our CB+ cells were also FoxP1+ (97.65% ± 2% in *n* = 2 males and 98.5% ± 2.5% in *n* = 1 female; white arrows in **Figures 2D–F**). These results classify these cells as MSNs. The few heavily labeled CB+ cells dispersed in the striatal region were likely interneurons (white arrow head in **Figures 2D–F**), like in the mouse striatum. In the areas poor in CB+ cells we also found that some of them co-localized with FoxP1 (data not shown).

In summary, we detected CB+ MSNs in male and female budgerigar striatum, located mainly in the medial regions of the striatal domain, with the exception of the vocal nucleus MStm that lacks this cell type, in contrast to the striatal vocal nucleus Area X in zebra finch.

### CB+ MSNs IN QUAIL STRIATAL DOMAIN AT ADULT STAGES AND DURING DEVELOPMENT

**FIGURE 3:**
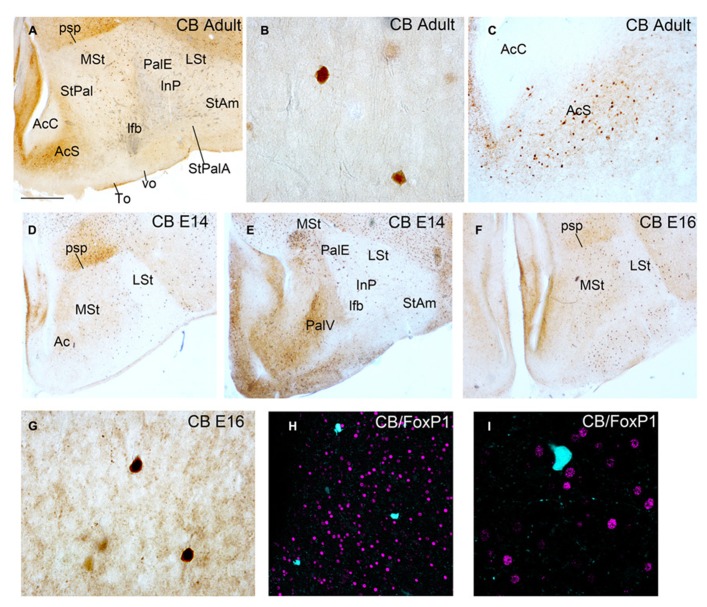
**Coronal sections through the striatum of adult quail (A–C) and quail embryos at E14 and E16 (D–I) showing CB immunostaining (A–G) and double immunofluoresecence of CB in cyan and FoxP1 in magenta (H,I).** The respective staining and stage are indicated at the upper right-hand corner of each panel. Dorsal is oriented toward the top of the photos, medial (bordering the ventricle) to the left. **(H,I)** show no co-localization of CB+ cells with FoxP1+ cells. For abbreviations, see list. Scale bar = 2 mm in **(A,D–F)**; 62.5 μm in **(B,G)**; 0.25 mm in **(C)**; 65 μm in **(H)**; 31.25 μm in **(I)**.

We analyzed the presence of CB+ cells in the striatal domain of adult male quail (*n* = 1) and quail embryos (*n* = 3) at different developmental stages (**Figure 3**). In adult male quail a short number of heavily stained CB+ cells appeared sparsely distributed in the striatal domain, from rostral to caudal levels, including the medial and lateral striatum (MSt and LSt), the StAm and the StPal regions with the InP included (**Figures 3A–C**). This pattern of CB expression was similar to the previously published CB immunostaining of chicken striatum (Bálint and Csillag, 2007). In the shell region of the nucleus Ac (AcS) we observed an increase in extracellular CB immunoreactivity accompanied by an increase in the number of CB+ somata, when compared with other striatal regions (**Figures 3A,C**). In contrast, the accumbens core region (AcC) was devoid of CB immunoreactive perikarya (**Figures 3A,C**). This result was also similar to the data from Bálint and Csillag (2007) in the nucleus accumbens of adult chicken. We checked the type of CB+ cell observed in adult quail striatum studying the co-localization with FoxP1 in the striatal domain, as we previously did in budgerigar. We did not detect co-localization of the two proteins neither in adult male (data not shown) nor at developmental stages (**Figures 3H,I**). This result indicated that CB+ cells in quail male striatum did not correspond to the MSN type described in budgerigar (present results).

Garcia-Calero and Scharff (2013) did not detect CB+ MSNs in the striatal domain of zebra finch females from PHD20 onward. However, during development, female zebra finches had CB+ MSNs in the striatal domain similar to males. To figure out if this is also the case in quail, we analyzed the presence of CB+ cells in the striatal domain of quail at different developmental stages (E14, *n* = 1 and E16, *n* = 2: **Figures 3D–I**). We also studied the co-localization of these cells with the MSN marker FoxP1 (**Figures 3H,I**). We obtained a similar pattern of CB immunostaining between embryos and adult quails in most of the striatal domains. There was no co-localization of CB and FoxP1 in the striatal region (**Figures 3H,I**).

In conclusion, we detected a few CB+ cells in the striatal domain of quail at adult and developmental stages. These cells appeared sparsely distributed and did not co-localize with FoxP1, suggesting that these cells were not MSNs.

### THE CB+ STRIATAL CAPSULE IN BIRDS

**FIGURE 4:**
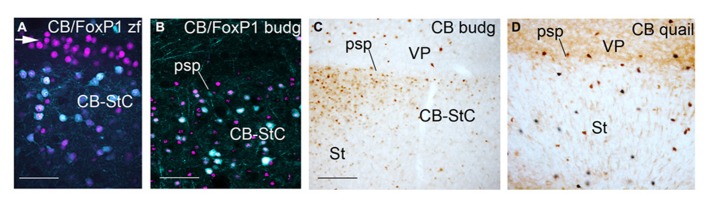
**Pallial/subpallial border in zebra finch at PHD12 (A), adult male budgerigar (B,C) and adult quail (D) showing double immunofluorescence of CB in cyan and FoxP1 in magenta (A,B) and CB immunostaining (C,D).** Dorsal is oriented toward the top of the photos, medial (bordering the ventricle) to the left. The white arrow in **(A)** points to the CB negative, Foxp1 positive domain over the CB+ striatal capsule. For abbreviations, see list. Scale bar = 62.5 μm in **(A)**; 65 μm in **(B)**; 0.25 mm in **(B,D)**.

A CB+ striatal capsule was previously described in male and female zebra finches (Garcia-Calero and Scharff, 2013). This structure, underlying the pallial/subpallial boundary, developed at early postnatal stages and contained a dense population of CB+ MSNs. This solid band of CB+ cells co-localized with DARPP-32, FoxP1, and FoxP2 and was located under a CB negative but DARPP-32/FoxP1/FoxP2 positive striatal belt (Figure 3G in Garcia-Calero and Scharff, 2013; white arrow in **Figure 4A**, present results; and data not shown for FoxP2). These authors suggested that the striatal capsule described in chicken by Puelles et al. (2007) consisted of two bands perpendicular to the pial surface in the striatal domain of zebra finch. In mammals, a CB+ striatal capsule is described at least during developmental stages (Liu and Graybiel, 1992a). In this work we analyzed the existence of a CB+ striatal capsule in the two birds species studied: budgerigar and quail. We also show CB and FoxP1 staining in the pallial/subpallial border of male zebra finch at PHD12 for comparative purposes.

In adult male/female budgerigars, we observed a CB+ striatal capsule in the medial and lateral striatal regions close to the limit with the pallium (adult male: **Figures 4B,C**). This capsule is composed of CB+ MSNs (CB+/FoxP1+ cells) like the ones described in zebra finch, but the cells were not as densely packed as in songbirds. In addition, we did not find a CB negative but FoxP1+ striatal capsule (white arrow in **Figure 4A**) dorsally to the CB+ FoxP1+ band, as seen in zebra finch (compare **Figures 4A,B**). Finally, in adult and developing quail embryos a CB+ striatal capsule was not observed and only dispersed CB+ positive cells with high CB-immunoreactivity were detected in the boundary with the pallial domain (**Figure 4D**).

In conclusion, a CB+ striatal capsule was observed in budgerigar striatum, similar to the one found in zebra finches. This structure was not detected in quail. A CB negative but FoxP1 positive lamina overlaying the CB+ striatal capsule was not observed in budgerigar, in contrast to zebra finch.

## DISCUSSION

The main goals of the present study were: (a) A description of the CB+ MSN population in the striatal domain of male and female budgerigars (vocal learner birds) in contrast to quail (a non-vocal learner bird); (b) To analyze the lack of this cell type in the striatal nucleus for song learning MStm in budgerigars in contrast to zebra finches; (c) the description of a CB+ striatal capsule in vocal learner but not in non-vocal learner birds.

### CB+ MSNs IN VOCAL-LEARNER BIRDS VS. NON-VOCAL LEARNER

Budgerigars and zebra finches are able to learn sounds by imitations, contrary to other birds like chicken or quail that are only able to produce innate sounds. Parrots are also capable of complex motor learning like movement learning by imitation (Moore, 1992). These differences in learning capabilities are also reflected, for example, in the existence of a song system in vocal learners in contrast to non-vocal learner birds (Nottebohm et al., 1976; Gahr, 2000). In the present work, we described a new significant difference in the striatal domain of vocal learner and non-vocal learner birds: CB+ MSNs were widely distributed in the medial striatal domain of male and female adult budgerigars, whereas, there was no production of CB+ MSNs in quails at developmental stages or in the adult.

Previous studies reported the existence of CB+ cells in the striatal domain of non-vocal learner birds like chicken (i.e., Bálint and Csillag, 2007; Husband and Shimizu, 2011), however, these studies did not define the type of neurons (interneuron or projecting neuron). In our work, CB+ cells are dispersed in the quail striatal domain, show high CB-immunoreactivity and do not co-localize with the MSN marker FoxP1. We propose that this CB+ cells are likely interneurons. In addition, a CB+ MSN population was described in songbirds striatum located mainly in the nucleus for song learning Area X (Garcia-Calero and Scharff, 2013). These results show important differences in the cytoarquitecture of the striatal domain between vocal learner and non-vocal learner birds that could relate with differences in neurofunctional capabilities like vocal learning or movement learning by imitation. This correlates directly with the role that CB protein could play in this neuron type (see below).

As we have mentioned before, the striatal domain in mammals is subdivided in striosome and matrix compartments. The striosome subdivision appears early during striatal development; the matrix compartment (the one containing the CB+ MSN population) is produced later during development from the SVZ (van der Kooy and Fishell, 1987; Anderson et al., 1997; Garel et al., 1999; Mason et al., 2005). Mutations of genes important for the development and functionality of the SVZ, like Dlx1/Dlx2 and Ebf1, generate abnormalities in the differentiation of matrix neurons and impair in CB+ MSN production (Anderson et al., 1997; Garel et al., 1999). These findings indicate that CB+ MSNs originate from the striatal SVZ and show a late neurogenesis. A SVZ in the striatal domain is also described in birds (Striedter and Charvet, 2008; Charvet and Striedter, 2009). Parrots show an important expansion of the SVZ at embryonic and post-hatching stages and that correlates with a delay and expansion in time of neurogenesis and telencephalic enlargement (Striedter and Charvet, 2008). Moreover, the SVZ is thicker in embryonic parrots than in age-matched quails (Striedter and Charvet, 2008). In addition, hatchling zebra finches have a large SVZ similar in thickness and extent to that of parrots (Charvet and Striedter, 2009). Taken together, these results could explain the presence of CB+ MSNs in parrots and zebra finches in contrast to quails. Moreover, CB+ MSN production was observed in the male zebra finch at postnatal stages, which indicates a late neurogenesis for this cell type in songbirds (Garcia-Calero, unpublished observations). In this sense, the well noticed neurogenesis delay in the SVZ of parrots and songbirds in contrast to non-vocal learner birds could translate directly in the production of a new cell type (CB+ MSNs): shifts in neurogenesis timing could provide be the basis for CB+ MSN production in birds.

It would be interesting to know the possible role that the CB+ MSNs play in the striatal domain of budgerigar and zebra finch. CB protein in mammals is linked to rapid regulation of intracellular calcium levels critical for synaptic plasticity, a cellular process underlying learning and memory (reviewed in Schwaller et al., 2002). New studies focused on the function of CB protein and CB+ MSN in the process of motor learning in budgerigar and zebra finch could be interesting to assess the implication of this cell type in the acquisition of new intellectual capabilities during bird evolution.

To sum up, changes in CB+ MSN production among bird groups could be a consequence of differences in SVZ development and function. In this sense, quails do not produce CB+ MSNs in the striatal domain, in contrast to parrots and male and female zebra finches. The role of CB+ MSNs in complex motor learning process must be revisited.

### CB+ MSNs IN VOCAL LEARNER BIRDS

As we mentioned before, parrots and songbirds are able to learn sounds by imitation and to accomplish this, they have developed a special system for vocal acquisition (Paton et al., 1981; Brauth et al., 1994; Striedter, 1994; Heaton and Brauth, 1999; Jarvis and Mello, 2000; Roberts et al., 2002). Recently, Suh et al. (2011) showed by retrotransposon analysis that parrots are the closest living relatives of passerine birds. Studies suggest that vocal learning existed in a common ancestor of parrots and songbirds and that the neural system for vocal acquisition could be homologous between both orders (Suh et al., 2011). In this sense, MStm in budgerigar striatum is potentially homologous to zebra finch Area X. On the other hand, hodological studies on the bird song system (i.e., Striedter, 1994; Durand et al., 1997) have shown that the connectivity pattern of parrots and songbirds song nuclei differs, and that probably the song system is not homologous between both orders. In the present work we described the allocation of a CB+ MSN population in budgerigar striatal area. These cells were distributed mainly in the periventricular region of the striatal radial domain. The lateral stratum only showed dispersed CB+ cells, with the exception of the superficial olfactory tubercle. In addition, the MStm was poor in CB+ MSNs. This result contrasts widely with previous data published by Garcia-Calero and Scharff (2013) in zebra finch, which locate CB+ MSNs solely in the Area X and CB+ striatal capsule of juvenile and adult males. Therefore, budgerigars and zebra finches are able to produce CB+ MSNs but the allocation of this cell type in the striatal domain changes among species.

The distribution of CB+ MSNs in male and female adult budgerigars parallels the data obtained in female and male zebra finches at early stages. CB+ MSNs distribute widely in the rostral part of the medial striatum at developmental stages, both in male and female zebra finches (i.e., Figures 1A,B,F,L–N, 2A,B, and 5A–E in Garcia-Calero and Scharff, 2013), while at the same time the lateral striatum only show dispersed CB+ cells (Garcia-Calero, unpublished observations). During the course of striatal development these cells gradually disappear in the medial striatal region except in the Area X and the CB+ striatal capsule in males and the latter in females. Due to the evident sexual dimorphism in CB+ MSN distribution in zebra finch striatal domain from PHD12, Garcia-Calero and Scharff (2013) proposed that these results could relate with estrogenic effects on Area X, mediated transynaptically via the HVC-to-Area X afferents arrival at early juvenile stages (Gahr, 1990; Mooney and Rao, 1994; Foster and Bottjer, 1998). This means that CB+ MSNs are located at a similar position (medial striatum, close to the proliferative regions) in male/female zebra finches at early stages and male/female adult budgerigars, however in zebra finches the HVC-inputs arrival triggers a re-allocation of this cell population. The song system in budgerigar has been well described and its connectivity pattern analyzed previously (Striedter, 1994; Durand et al., 1997). Budgerigars show important similarities in song nuclei distribution to zebra finch. However, the comparable nucleus to HVC in parrots, the central nucleus of the lateral nidopallium (NLC), does not project directly to the MStm, but through an intermediate relay in a mesopallial nucleus (Striedter, 1994; Durand et al., 1997). If HVC-to-Area X connectivity is the cause of CB+ MSNs re-allocation and maintenance in male zebra finch, a different connectivity pattern of MStm nucleus in budgerigar could explain our present findings.

### CB+ STRIATAL CAPSULE IN VERTEBRATE DEVELOPMENT AND EVOLUTION

A CB+ striatal capsule was described in detail by Garcia-Calero and Scharff (2013) in male and female zebra finches from PHD5 onward. This neuroanatomical structure laid in the pallial/subpallial boundary, forming a dense cellular band of CB+ cells that extended from the ventricular zone to the striatal surface. The CB+ cells co-localized with DARPP-32, FoxP1, and Fox2, three markers for MSNs. A DARPP-32/FoxP1/FoxP2 positive, CB negative domain overlies this CB+ strip (Figure 3G in Garcia-Calero and Scharff, 2013). Puelles et al. (2000,2007) has previously described a striatal capsule in chicken at developmental and adult stages. In adult chicken this structure appeared differentially labeled in the AChE and TH stainings, and showed patchy reaction particularly subpially. Haesler et al. (2004) also detected a FoxP2+ band forming thicker clumps of FoxP2 positive cells in the interface of the pallium and subpallium of adult male zebra finches. At chicken developmental stages a thin band of Pax6+ cells, lying bellow the pallial/subpallial border, was described by Puelles et al. (2000) and Abellan and Medina (2009). Abellan and Medina (2009) suggested that the striatal capsule is a derivative of this Pax6+ dorsal-most striatal domain in chicken and the same region in mouse produced the intercalated nucleus of the amygdala. However Garcia-Calero and Scharff (2013) observed the CB+ striatal capsule located in the ventral Islet1+-Pax6 negative striatal domain in zebra finches. In rats, Liu and Graybiel (1992a) described a transient population of CB+ cells located in the boundary of the striatal domain with the pallium at early postnatal stages. It is evident that there is not a unified criterion about what we call striatal capsule in vertebrates, and this situation complicates the study of homologies among species and the understanding of its functional meaning. In our study, we detected a CB+ striatal capsule in adult budgerigar, even in the CB-poor lateral domain. The CB+ cells co-localized with FoxP1, but the distribution was sparser than in zebra finch. In addition, a CB-negative, DARPP-32/FoxP1/FoxP2 positive band overlaying the CB+ striatal capsule was solely described in zebra finch, whereas a similar structure was not evident in budgerigar. CB+ MSNs were not described in the striatum of quail at adult and developmental stages. Moreover, a CB+ striatal capsule was not found at any of the stages analyzed in these birds. For all these reasons, to understand the function and evolution of this region, it would be necessary to define what we call striatal capsule in vertebrates. In the present report and previous ones (Puelles et al., 2007; Garcia-Calero and Scharff, 2013) the striatal capsule is defined differently according to the species analyzed: a radial structure subdivided in a CB negative and a CB positive bands in zebra finch; a simple CB+ band at the boundary of the pallial domain in budgerigar and mouse; and an AChE/TH+, CB negative domain in adult chicken. In addition, the present results show important molecular differences in the pallial/subpallial boundary among bird species, pointing to patterning diversity during evolution of this region.

In conclusion, in the present work we described important differences in the distribution and type of CB+ cells in the striatum of representative species of vocal learner and non-vocal learner avian orders (respectively the budgerigar and the quail). In addition, we observed differences in the CB+ MSNs allocation between close vocal learner bird families.

## Conflict of Interest Statement

The authors declare that the research was conducted in the absence of any commercial or financial relationships that could be construed as a potential conflict of interest.

## AUTHOR CONTRIBUTIONS

All authors had full access to all the data in the study and take responsibility for the integrity of the data and the accuracy of the data analysis. Conceived and designed the experiments: Elena Garcia-Calero and Salvador Martinez Performed the experiments: Elena Garcia-Calero and Olga Bahamonde Analyzed the data: Elena Garcia-Calero and Salvador Martinez Wrote the article: Elena Garcia-Calero and Salvador Martinez Obtained funding: Elena Garcia-Calero and Salvador Martinez.

## Abbreviations

Ac: accumbens nucleus
ac: anterior commissure
AcC: accumbens nucleus, core region
AcS: accumbens nucleus, shell region
budg: budgerigar
CB: calbindin
CB-StC: Calbindin-positive striatal capsule
E14, E16: embryonic day 14, 16
HA: apical hyperpallium
InP: intrapeduncular nucleus
lfb: lateral forebrain bundle
LSt: lateral striatum
MD: mesopallium, dorsal part
MSt: medial striatum
MStm: magnocellular nucleus of the medial striatum
MV: mesopallium, ventral part
N: nidopallium
PalE: ectopic pallidum
PHD: posthatch day
psp: pallial-subpallial boundary
St: striatum
StAm: striatoamygdaloid transition area
StC: striatal capsule
StPal: striatopallidal area
StPalA: striatopallidal amygdaloid area
To: olfactory tubercle
vo: ventral olfactory tract
VP: ventral pallium.
